# Joint temporal trends in river thermal and hydrological conditions can threaten the downstream migration of the critically endangered European eel

**DOI:** 10.1038/s41598-021-96302-x

**Published:** 2021-08-19

**Authors:** Elorri Arevalo, Hilaire Drouineau, Stéphane Tétard, Caroline M. F. Durif, Ola H. Diserud, W. Russell Poole, Anthony Maire

**Affiliations:** 1 INRAE, Unité EABX – Écosystèmes Aquatiques et Changements Globaux, HYNES (INRAE-EDF R&D), 50 avenue de Verdun, 33612 Cestas Cedex, France; 2 ICEO Environnement, 52 Ter avenue des Sables, 85440 Talmont-Saint-Hilaire, France; 3 EDF Recherche et Développement, Laboratoire National d’Hydraulique et Environnement, HYNES (INRAE-EDF R&D), 6 quai Watier, 78401 Chatou Cedex, France; 4 Institute of Marine Research, Austevoll Research Station-Saugeneset 16, 5392 Storebø, Norway; 5 Norwegian Institute for Nature Research, Torgarden, P.O. Box 5685, 7485 Trondheim, Norway; 6 Marine Institute, Furnace, Newport, Co. Mayo Ireland

**Keywords:** Animal migration, Behavioural ecology, Climate-change ecology, Conservation biology, Freshwater ecology

## Abstract

Climate change is modifying the hydrological and thermal regimes of rivers worldwide, threatening the triggering of organisms’ key life-cycle processes. European eel (*Anguilla anguilla*) is a critically endangered fish species that migrates over several thousand kilometres between its rearing habitats in continental waters of Europe and North Africa and its spawning area in the Sargasso Sea. Downstream migration of adult eels occurs during periods of decreasing river water temperature associated with high discharge but changes in these environmental cues may affected eel migratory conditions. An innovative multivariate method was developed to analyse long-term datasets of daily water temperature, discharge and eel passage in two European rivers. Over the past 50 years, water temperature and discharge increased in both rivers during the downstream migration period from August to November. Silver eels preferentially migrated at temperatures between 10 and 20 °C combined with high discharge. Environmental changes have resulted in the migration of silver eels under warmer water temperatures. This example illustrates how the changes in environmental cues have led to a growing mismatch between the migratory conditions preferentially selected and those actually used, which may threaten the completion of the eel’s life cycle and ultimately the persistence of this already critically endangered species.

## Introduction

Every year, billions of animals migrate seasonally to find food or mates, avoid predators or escape unsuitable weather conditions^1^. Many migratory species, including diadromous fish, are in decline due to various components of global change such as overexploitation, climate change, habitat loss due to anthropogenic barriers or land-use changes^2,3^. Global change threatens ecological processes like animal migrations, which are synchronized by complex interactions of environmental cues. In many rivers, climate change has resulted in increased water temperature^4^ and has affected seasonal precipitation levels^5^ causing changes in hydrological cycles. Thermal and hydrological regimes are also altered by water abstraction for human consumption, irrigation, industry and hydropower production^6^. The asynchronous modification of thermal and hydrological regimes is a threat for many species whose migration is triggered by thermal and hydrological cues^7,8^. To illustrate this, let’s assume that a species needs a specific water temperature to migrate and that due to global change such temperature is occurring earlier in the year. Among possible adaptive responses, the species could migrate earlier to stay in phase with the temperature (change in phenology), or migrate at the same period but adapt to the new warmer conditions (local adaptation)^9^. But now, let’s assume that the species simultaneously needs suitable river discharge to migrate and that, because of global change, the suitable discharge occurs later in the year. Then, the species would have to migrate later to have suitable discharge, but earlier to have suitable water temperature. This example illustrates that, when an ecological process is triggered by two environmental factors affected by global change, the question is not only whether the species will undergo the process earlier or later, but whether the suitable joint conditions still occur.

The European eel (*Anguilla anguilla*) is a catadromous species that migrates over several thousand kilometres. Adults spawn and die in the Sargasso Sea and larvae, called leptocephalus, are transported by ocean currents over 6000 km to the coasts of Europe and North Africa^10^. After reaching continental shores, larvae transform into glass eels, enter continental waters where they turn into yellow eels and grow generally for 5–30 years or more^11,12^. Then, adult eels eventually undergo a final metamorphosis during summer^13^, called silvering, which is a pre-adaptation to the marine environment^14^. Once silvering is completed, downstream migration from rivers to their marine spawning grounds occurs during periods of favourable environmental conditions^15^ typically from July to January in the north of its distributional range and slightly later (from October to March) in the southern and eastern Mediterranean^16^. The combination of a number of factors, such as day length, light conditions during the dark hours (*i.e.*, moon phase), water temperature and river discharge^13,17^ may influence the preparation of silver eels to migrate. These factors may interact changing the migration activity in different ways^18^. This paper focuses on the effect of two major factors: river temperature and discharge, in the light of global climate change.

Global change has led to a rapid decline in European eel populations^19^ and the species is listed as critically endangered by the IUCN^20^. In this context, we explored whether the joint evolution of river water temperature and discharge over the last decades has affected silver eel migration. In some rivers, global warming has induced modifications in the thermal regime that have had a visible impact on the seasonal migration pattern of silver eel^18^. Meanwhile, higher precipitation levels in northern Europe are expected to increase river discharge in summer^21^ and advance migration while, conversely, drought periods in southern Europe are expected to extend into fall^22,23^ and delay migration. These regional contrasts make it difficult to predict the consequences of changing climate on the migration dynamics of eels. Moreover, how eels perceive changes in water temperature and discharge is still unclear. Water temperature likely affects eel physiology and the silvering (maturation) process, while discharge acts as an immediate trigger either directly through mechanoreceptors located on the lateral line of individuals, or indirectly by increasing the water turbidity, which in turn can be perceived by the sensory organs^13,14,24^. Overall, temperature regulates eel physiological readiness for migration while discharge facilitates their downstream movements towards the sea. However, even if eels are physiologically ready to migrate, insufficient discharge may lead to postponed migration. Thus, a mismatch in the evolution of these two environmental factors may disrupt eel migration mechanisms. More specifically, in the event of climate change leading to raised temperatures, and differing availability of higher water discharge, not only the historical timing, but also the frequency of occurrence of the combination of these may be changing. This paper examines the co-evolution of these two factors and the continued availability of this combination in the light of changing conditions. The immediate question is whether the historical combinations of optimum water temperature and discharge continue to occur, and if environmental changes have resulted in changes in the conditions effectively used by eels to migrate. Analysing the co-evolution of two joint environmental factors is not a straightforward task and thus a new statistical method had to be developed to detect joint trends in multivariate time-series^8^. However, a methodological gap persisted in highlighting trends in environmental conditions and quantifying the consequences of such trends on phenological events^25^. We propose an additional and complementary method to fill this gap by considering simultaneously the occurrence and the intensity of a life-cycle event through weighting environmental conditions by daily counts of migratory eels. Specifically, we defined the ‘available niche’ as the water temperature × discharge conditions potentially available for eel migration. Within this available niche, we quantified eel migration preference (‘preferential niche’) which indicates whether an available condition is preferentially selected or rejected. We defined the ‘effective niche’ as the conditions in which the eel migration had effectively occurred (*i.e.*, a condition can be rejected by most eels but used by at least one individual to migrate and thus become part of the effective niche). To explain these different niches, we can make a parallel with the concepts of supply and demand: available niche would correspond to the supply and preferential niche to the demand. The effective niche would then be the result of the interaction between supply and demand.

The method was applied to two European rivers (the Imsa River in Norway and the Burrishoole River in Ireland) where water temperature, discharge and silver eel migrations have been monitored daily since the 1970s. We aimed to (1) investigate changes in environmental conditions (*i.e.*, the ‘available niche’) over the study period, (2) examine changes in the ecological niches of silver eel (*i.e.*, the ‘effective’ and ‘preferential’ niches), and (3) discuss the implications of potential niche shifts.

## Material and methods

### Study sites and data used

**Figure 1:**
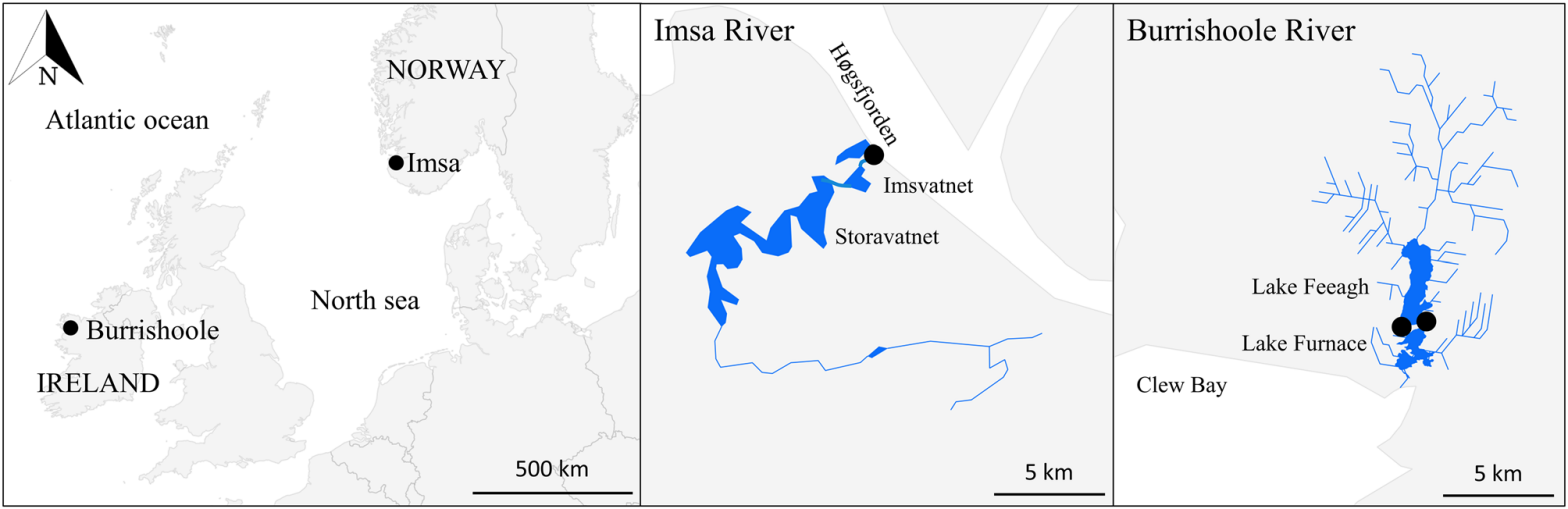
Location of the monitoring stations (●) for water temperature, discharge/water level and eel counting on the Imsa River in Norway and on the Burrishoole River in Ireland.

The Imsa watershed is located in southwestern Norway (58° 54’ N, 5° 57’ E) and covers an area of 128 km^2^ (Fig. 1). The watershed is sparsely urbanised (1.5% of the total surface of the watershed) and dominated by natural areas (54.8%) and agriculture (43.8%)^26^. The hydrology of the Imsa River is mostly influenced by rainfall, with a gradual decrease in discharge from January to June, followed by a period of low flow in summer and an increase in fall (see Supplementary material, Fig. S1). A Wolf-type fish trap is located about 100 m upstream of the river mouth and has been operating throughout the year since 1975 to catch fish during their downstream migration. The distance from the trap to the upper limit of eel’s suitable habitat in the Imsa watershed is approximately 20 km^18^. Water temperature and discharge have been recorded daily since 1975. The data considered in the present study covered the period 1975–2019. Mean annual water temperature in the Imsa River varied between 7.9 and 10.7 °C (interannual average = 9.4 °C) and mean annual discharge varied between 3.5 and 7.6 m^3^ s^−1^ (interannual average = 5.2 m^3^ s^−1^).

The Burrishoole watershed is located in western Ireland (53° 56’ N, 9° 35’ W) and covers an area of 90 km^2^ (Fig. 1). The watershed is dominated by natural areas (*i.e.*, forests, peat soils and wetlands and lakes), sparsely populated and the main land-use is coniferous forestry and subsistence agriculture^27^. The hydrology of the Burrishoole River is spatey and mainly influenced by rainfall. Two upstream and downstream Wolf-type fish traps are located on two outflow rivers from Lake Feeagh to the brackish Lake Furnace and have been operating year-round since 1971^28,29^. The distance from the traps to the upper limit of eel’s suitable habitat in the Burrishoole watershed is approximately 13 km^18^. In this study, water level in Lake Feeagh was used as a proxy for water discharge^18^. Due to major modifications in the monitoring of the lake water level in 2013, subsequent data were discarded to avoid potential bias. Water temperature and water level have been recorded daily since 1970. The data considered in the present study covered the period 1971–2013. Mean annual water temperature in the Burrishoole River varied between 9.2 and 12.4 °C (interannual average = 10.5 °C) and mean annual water level varied between 0.33 and 0.49 m (interannual average = 0.41 m). Annual and seasonal means of water temperature and discharge/water level are provided in Table S1 for both rivers.

**Figure 2:**
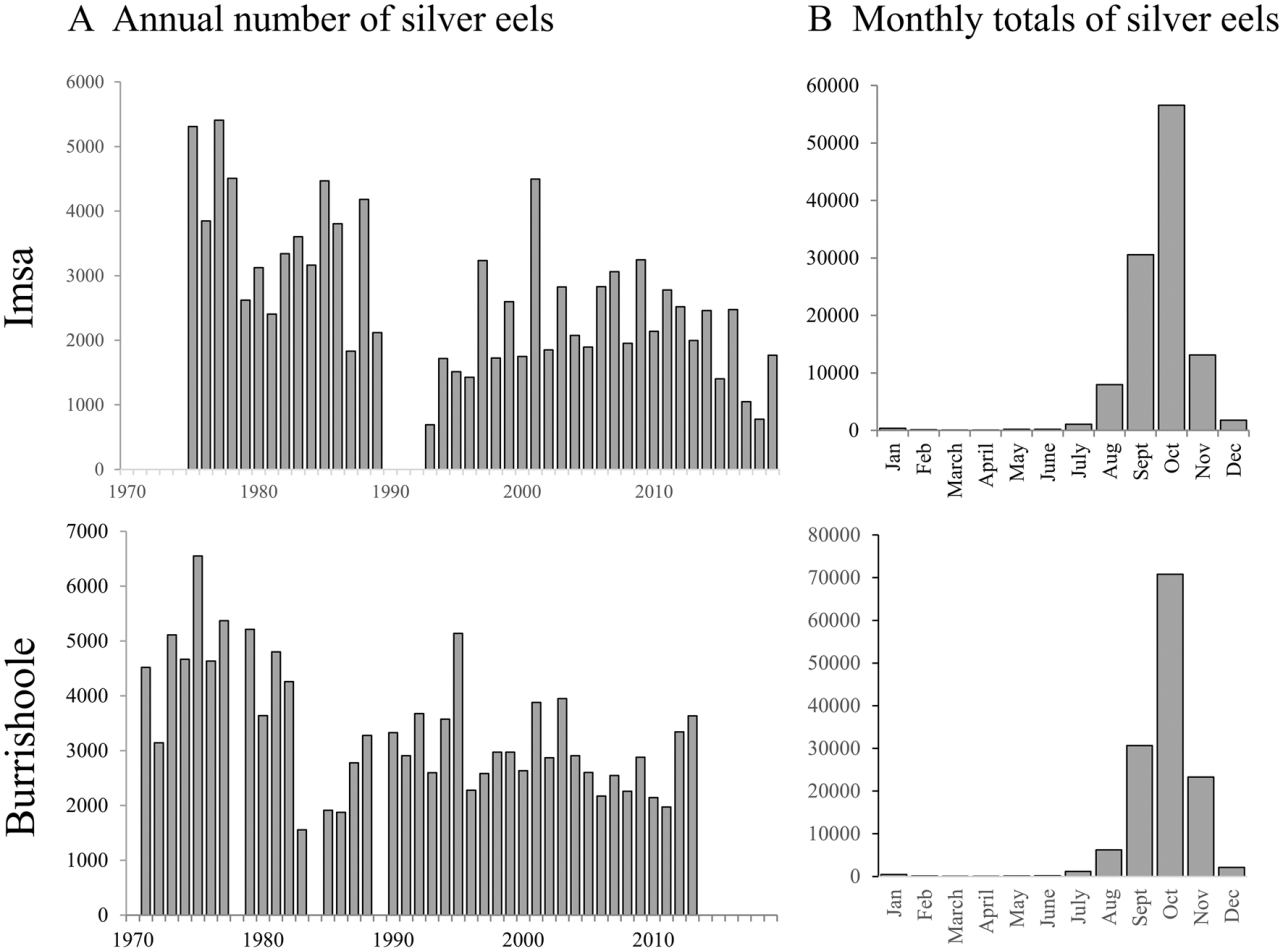
Description of silver European eel (*Anguilla anguilla*) populations migrating on the Imsa and Burrishoole rivers: (**A**) Annual number of silver eels caught and (**B**) total catch per month over the study period. Mann–Kendall trend tests were performed on annual catches (Mann–Kendall tau = − 0.33 and *p*-value = 0.01 on the Imsa River; Mann–Kendall tau = − 0.35 and *p*-value = 0.01 on the Burrishoole River).

In both rivers, the traps are checked once or twice every day throughout the year. Catches were summed up to get the number of migrating silver eels per 24 h. Biological years were defined according to the migration period of silver eels^16^ (Fig. 2B), from the 1st of June of year N to the 31st of May of year N + 1. For the Imsa River, between 706 (in 1993) and 5,355 (in 1977) silver eels have been trapped yearly (interannual average = 2687 eels; Fig. 2A). For the Burrishoole River, between 1559 (in 1983) and 6,549 (in 1975) silver eels have been trapped yearly (interannual average = 3379 eels). Mann–Kendall trend tests were performed on annual number of silver eels caught to test for temporal trends over the study period.

### Trends in annual water temperature and discharge associations

Water temperature and discharge data were analysed using the Choc method^8^ to determine which associations of water temperature and discharge have become significantly more or less frequent over the years. First, yearly densities of probability were estimated with kernels at each pair of water temperature and discharge conditions. The bandwidth parameter (H), which defines the degree of smoothing of the kernel curves, was selected according to the plug-in method^30^. Second, Mann- Kendall trend tests were used on the densities of probability over the entire study period for each pair of water temperature and discharge conditions to detect monotonic temporal trend. Statistical significance was assessed by comparing results with those of artificial datasets generated under the null hypothesis, which implied that the frequency of a given water temperature and discharge association did not change over time. This approach was applied separately to Imsa and Burrishoole rivers for the full range of biological years available to reveal annual environmental trends. Discharge and water level were log-transformed prior to analyses for normalization purposes and to limit data over-dispersion.

### Trends in water temperature and discharge associations available during the downstream migration period: the available niche

The Choc method was applied to daily water temperature and discharge/water level data covering the migration season (from August to November; Fig. 2B), which represented the key time period when silver eels experience possible environmental changes, to detect trends in the available niche, *i.e.*, the occurrence of water temperature × discharge associations during the period of silver eel migration. Kernels estimated during the migration period were denoted *Kde*_*env*_.

### Trends in water temperature and discharge associations effectively used by silver eels during downstream migration: the effective niche

The daily proportion of migrating silver eels was calculated per biological year as the daily count divided by the total counts of the biological year. To detect whether changes in the available niche have led to changes in the environmental conditions effectively used for downstream migration, *i.e.*, the ‘effective’ niche, we applied another Choc analysis on water temperature and discharge data restricted to the migration season (similarly to the available niche) with the exception that days were weighted by their daily proportion of migrating silver eels. Kernel estimates weighted by the daily proportions were denoted *Kde*_*eel*_.

### Preferential niche of silver eel migration and its change over time: the preferential niche

To characterize the preferential niche for silver eel migration (*i.e.*, water temperature and discharge associations preferentially selected by eels to migrate), we built an electivity index based on the Ivlev feeding electivity index^31,32^, which was commonly used in gut content analyses to reveal prey preferences of predators. This index identified prey “selected” or “rejected” by a predator, following the standard terminology generally associated with diet analyses. Consequently, we adapted it to refer to the environmental conditions “selected” or “rejected” by silver eels for their downstream migration by taking into account, for each association, the frequency of a water temperature × discharge association in the environment, the occurrence of a migration event and the number of eels migrating under this association. The electivity of a water temperature × discharge association *E’(T,Q)* was calculated as follows:

$$ E^{\prime}\left( {T,Q} \right) = \frac{{\left[ {Kde_{eel} \left( {T,Q} \right) - Kde_{env} \left( {T,Q} \right)} \right]}}{{\left[ {Kde_{eel} \left( {T,Q} \right) + Kde_{env} \left( {T,Q} \right)} \right]}} $$

With *Kde*_*eel*_*(T,Q)* and *Kde*_*env*_*(T,Q)* the estimated kernel densities of probability of occurrence of water temperature *T* and discharge *Q* association estimated on the basis of the effective and available niches, respectively. Electivity index values close to -1 corresponded to associations “rejected” by silver eels whereas values close to 1 indicated preferentially “selected” associations. As index values were highly fluctuating and not reliable for low kernel estimations, the electivity index was not calculated for the 5% rarest environmental associations. These conditions corresponded mostly to periods of low discharge that are not suitable for silver eel migration.

**Figure 3:**
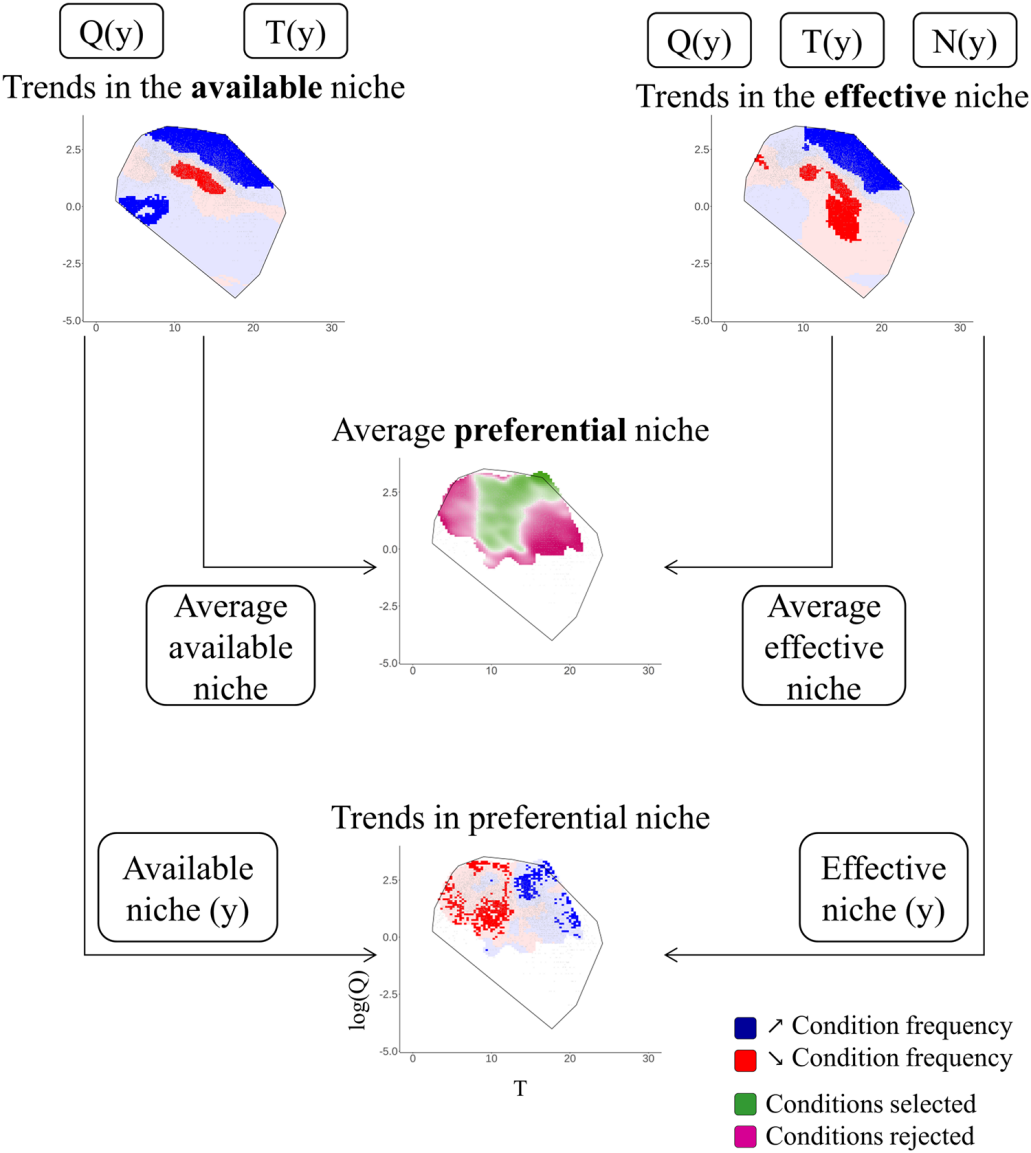
Schematic representation of the methodological framework and links between the performed analyses. Daily water temperature *T* and discharge *Q* are used to identify trends in the available niche over the years *y*. *T* and *Q*, weighted by the daily proportions of eel passage *N*, are then used to identify trends in the effective niche. The available and effective niches are finally used to calculate an average preferential niche over the study period and trends in the preferential niche over the years *y*.

To get an overall picture of the preferential niche over the whole study period, electivity indices were first computed using kernel density estimations fitted on all years pooled together (*i.e.*, average preferential niche). Then, to investigate temporal trends in this niche, yearly electivity indices were computed for each water temperature × discharge association using yearly *Kde*_*eel*_ and *Kde*_*env*_. Then, similar to a Choc analysis, Mann–Kendall trend tests were performed on the electivity indices of each pair of associations. Statistical significance was assessed by comparing the results obtained on the observed time series with those from artificial datasets generated by permuting years randomly (resulting in artificial datasets without monotonic trend). A summary of the analyses performed, and their linkages is provided in Fig. 3. The methodology was implemented in the R software^33^ ‘chocR’ package^34^.

### Ethical statement

All methods involved in eel trapping and manipulation were carried out in accordance with relevant guidelines and regulations. Concerning the River Imsa trap, all experimental protocols are approved by the Norwegian Environment Agency (ref. 2011/16,460 ART-FF-SJ). Concerning the River Burrishoole trap, the Marine Institute complies with EU directive on animal welfare 2010/63/EU and the corresponding Irish directive SI No 543 of 2012. Additionally, the data collection operates under licence (Fisheries Acts 1959–2003) from the Department of Agriculture, Food and Marine and by permission of the Minister of Agriculture, Food and Marine.

## Results

The annual number of silver eels migrating each year decreased significantly on the Imsa River between 1975 and 2019 and on the Burrishoole River between 1971 and 2013 (Fig. 2A).

**Figure 4:**
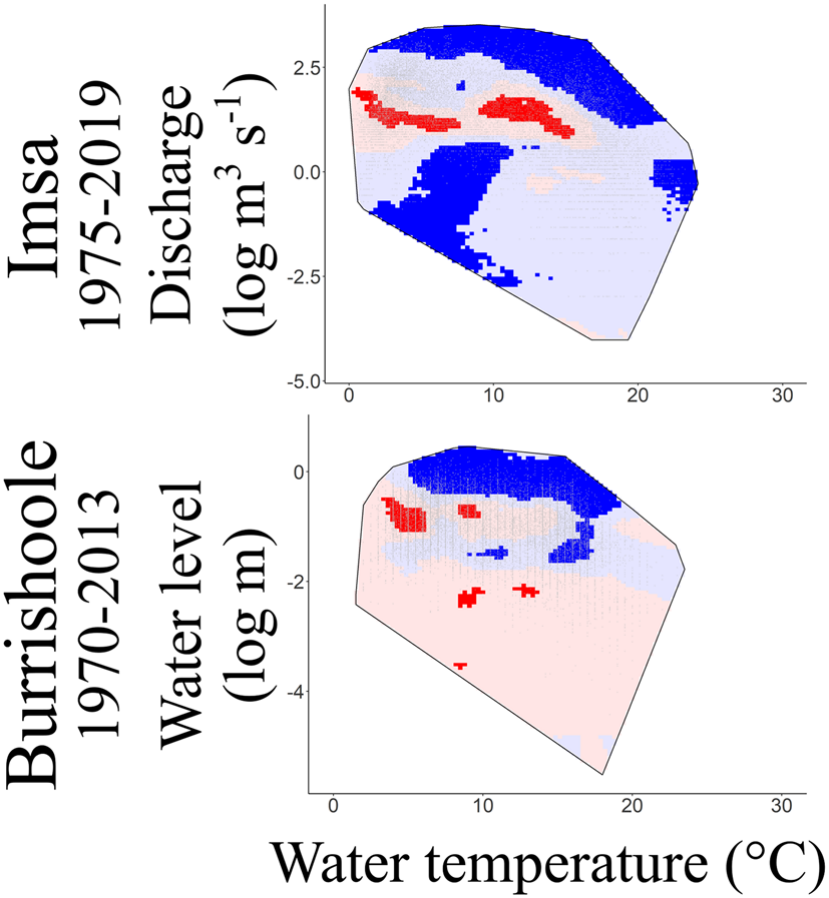
Two-dimensional heatmaps of water temperature (X-axis; °C) and log-transformed discharge/water level (Y-axis; log m^3^ s^−1^ or log m) associations for the Imsa and Burrishoole rivers at the annual scale. Water temperature × discharge/water level associations that have become more or less frequent over the study period are shown in blue or red, respectively. Light and dark colours correspond to non-significant and significant trends, respectively (e.g., dark red corresponds to significant decreasing trends, light blue to non-significant increasing trends). The grey line delineates the convex hull (*i.e.*, the smallest space encompassing all points of the annual dataset). See Fig. S2 for the results at the seasonal scale.

In the Imsa River, high discharge conditions have increased in recent years in comparison to the 1970s as well as days with low discharge (log-scale values of − 2.5–0; natural scale values of 0.1–1 m^3^ s^-1^) and cold temperature (< 10 °C) (Fig. 4). On the contrary, low and intermediate water temperatures (< 15 °C) associated with intermediate discharge (log-scale values of 1–2; natural scale values of 2.7–7.4 m^3^ s^−1^) have become less frequent than in the past. Similarly, in the Burrishoole River, high-water level occurrences have also increased since the 1970s (Fig. 4). These results apply to trend analyses at both annual and seasonal scales (but with respect to water temperature and discharge seasonal ranges; Fig. S2). Percentages of significant changes in the occurrence of environmental associations are provided in Table S2.

**Figure 5:**
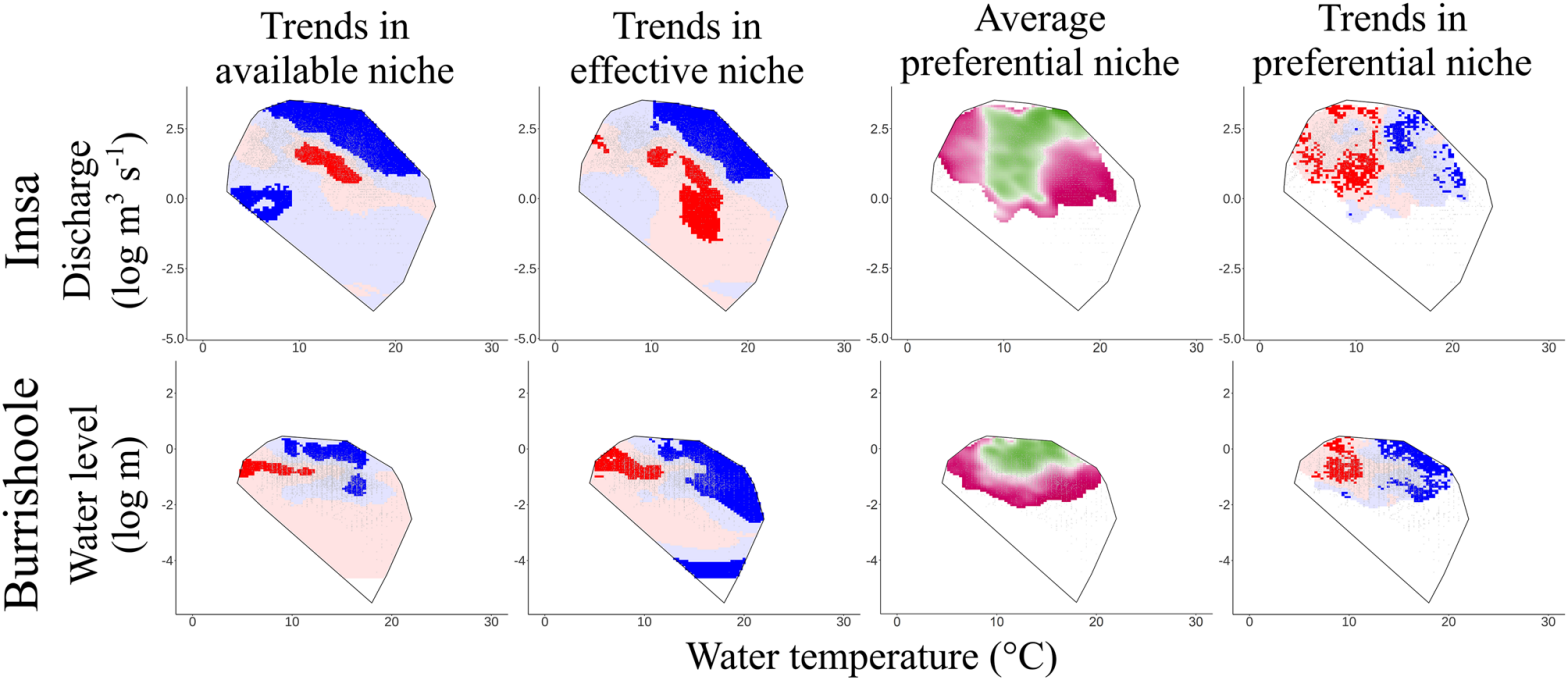
Migratory conditions of silver European eels (*Anguilla anguilla*) from the Imsa (1975–2019) and Burrishoole rivers (1970–2013). Two-dimensional heatmaps of water temperature (X-axis; °C) and log-transformed discharge/water level (Y-axis; log m^3^ s^−1^ or log m) are plotted for the period August to November (*i.e.*, the migration season) representing trends in the available niche, effective niche, average preferential niche and trends in the preferential niche. Discharge/water level and temperature associations that have become more or less frequent over the study period are shown in blue or red, respectively. Light or dark colours correspond to non-significant and significant trends, respectively. The grey line delineates the convex hull. Within the preferential niche, the 5% of the less frequent associations correspond to the white colour within the convex hull while the selected or rejected associations are shown in green or purple, respectively.

Concerning the migration season, occurrences of situations where water temperature was above 10 °C associated with high discharge have increased compared to the past (available and effective niches) on the Imsa River (Fig. 5). The average preferential niche corresponded to silver eels preferentially migrating at (1) water temperature between 10 and 15 °C, regardless of the discharge, and (2) water temperature between 15 and 20 °C with high discharge (Fig. 5). The preferential niche has remained essentially the same over time (see Fig. S3) but the selection of warm temperatures has increased compared to low temperatures (see trends in the preferential niche, Fig. 5).

In the Burrishoole River, water temperatures between 10 and 17 °C associated with high water level have increased during the migration season over the study period (*i.e.*, the available niche; Fig. 5). Trends in the effective niche were different from trends in the available niche: eels migrated more frequently than in the past at warm water temperatures (> 17 °C) or at intermediate water temperatures associated with low discharges. Similar to the Imsa River, silver eels preferentially migrated at water temperatures between 10 and 20 °C at high water level. Trends in the preferential niche demonstrated increased migration at high water temperature (> 17 °C) over the study period.

The results on these two rivers were similar: the main association within the available niche that became more frequent was warm temperatures associated with high discharges. These trends in the available niche drove totally (for the Imsa River) or partially (for the Burrishoole River) the trends within the effective niche. Preferential niches were also similar among rivers, with a selection of water temperature between 10 and 20 °C associated with high discharges, but this preference gradually tended towards warmer water temperatures.

## Discussion

In this article, we proposed an innovative method to link the occurrence of a species, or of a key life-cycle event, to various environmental cues simultaneously through the ecological niche concept^35^. Unlike traditional statistical methods, our method explicitly considers the correlation structure between the variables considered. This joint treatment of the variables is essential since the interaction between them may entail a differential impact on the ecological process of interest. For instance, a high discharge can trigger eel migration but the ecological consequences of this migration (such as energy costs) may not be the same depending on water temperature. We showed that in two watersheds located in the northern part of the geographic range of the European eel, high discharges were increasingly frequent, especially associated with warm water temperatures, both annually and during the core migration period (from August to November). We found that these trends in the frequency of environmental associations within the available niche led to a modification towards warmer water temperatures of the conditions used by silver eels to migrate.

Concerning the proposed approach, the effective and preferential niches can diverge: while eels preferentially “select” specific conditions, migration can occur in other conditions (even conditions identified as usually “rejected”) if the most suitable ones are not available. For example, we observed an increasing trend in the use of warm water temperatures in the effective niche on the Burrishoole River. However, this does not mean that these conditions are now the most frequently used by eels to migrate: these conditions were rare at the beginning of the study period and we detected an increase in the occurrence of such conditions compared to a near-absence at the beginning of the period. Additionally, we focus on the migration period (from August to November) because similar environmental conditions may occur at other times of the year but are not used by the eels because the migration peak has already occurred. Therefore, the eels' preference for a condition would be lowered if we considered the whole biological year. In our application of the Choc analysis, we assumed that eels display immediate responses to environmental conditions as we compared daily migrations to daily environmental conditions (though kernel smoothing reduces the immediacy). This is supported by the fact that the two watersheds studied are small and that changes in local conditions (e.g., precipitation, wind) have a very rapid effect on the thermal and hydrological conditions of the rivers. We therefore assumed that the initiation of their migration and capture by the trap occurred within one day.

The two 50-year datasets mobilized allowed us to robustly characterize the current effective and preferential niches for eel downstream migration. Using observations and measurements, we assessed past effects of global change on silver eel migration on the Imsa and Burrishoole rivers. A perspective of this work would be to further develop this method to allow the projection of possible effects of future hydroclimatic changes on the ecological niches for eel downstream migration, by combining it with climate and population dynamics models. Furthermore, to capture a more comprehensive picture of the effect of hydroclimatic trends on eel migration, it would be necessary to apply our approach to watersheds distributed throughout the species’ geographic range. However, despite multiple requests, we did not find any other long-term datasets that combined daily measurements of water temperature, river discharge and eel passage. Another limitation of our work was that the number of silver eels never became limiting by weighting the days with the daily proportions of annual migrants. We considered this assumption to be reasonable since we restricted the analysis to the core migration season, and the migrants appeared to be evenly distributed over from August to November. Future developments of the method could make it possible to weight observations by the daily number of migrating eels divided by the sum of remaining eels, with the risk of giving too much importance of late migratory episodes. The last migrants may be rather “in a hurry” to migrate, whatever the environmental conditions, which could have a significant impact on the preferential niche.

Global climate change has affected the rainfall-driven hydrological regimes of the Imsa and Burrishoole watersheds over the past 50 years. We detected an increase in the frequency of high discharge events on both rivers. This finding is consistent with studies that predict a severe alteration of natural flow regime by 2050^36^ with a significant increase in annual precipitation^37^ as well as in the annual number of rainy days^38^. The increase in precipitation level is expected to be considerable in winter (between + 15 and + 30%) while remaining moderate in summer (+ 5%)^36^. Extreme rainfall events are also expected to be more common and intense in small coastal watersheds^39^ similar to the Imsa and Burrishoole watersheds. Concurrently, water temperature increased on both rivers, as previously reported on the Imsa River over the last 20 years^29^ and on the Burrishoole River since 1960^40,41^. While global change is expected to have spatially heterogeneous effects on the hydrological regime of rivers (*i.e.*, increases in summer and fall discharges in northern Europe; decreases in southern and southwestern Europe^36^), the patterns observed over the last few decades were similar for the two rivers considered in this study.

The almost 50-year time-series from the Imsa and Burrishoole rivers provide a unique environmental baseline for assessing how global change has affected the environmental cues that regulate many ecological processes. Despite a decrease in the number of migrating silver eels^29^, there were enough individuals caught every year to study the environmental conditions associated with eel downstream migration. Given that the European eel is a panmictic species (*i.e.*, individuals originating from a single spawning area^42^) and environmental conditions are comparable within both rivers, the associations selected were unsurprisingly similar between the Imsa and Burrishoole rivers, with silver eels preferentially migrating at water temperature above 10 °C associated with high discharge. These preferentially selected conditions are consistent with existing observations^18,24^ but the possibility to migrate within a wide range, between 4 and 23 °C, has been observed^43^. Given that water temperature and discharge appear to be the predominant environmental cues triggering eel migration^15^, some other abiotic (e.g., photoperiod, barometric pressure, lunar phase or turbidity) or biotic factors (e.g., shoal effect^44^) interact to determine the intensity of migration^18^ and may explain individuals migrating under “rejected” conditions^45^. This wide range of water temperature can also be explained because the migration phenomenon is not only explained by the immediate environmental conditions, but also by the conditions experienced by eels in the months prior to migration^18^.

Water temperature and discharge conditions observed during the downstream migration period of eels (*i.e.*, August-November) have changed significantly on both rivers since the 1970s. Significant trends (either increases or decreases in frequency of occurrence) were detected in the effective niche for about 25% and 35% of all associations of water temperature and discharge conditions for the Imsa and Burrishoole rivers, respectively (see Table S2 for percentages of significant trends in each niche). We have shown an increased preference towards water temperatures above 15 °C and niche margins (*i.e.*, potentially suboptimal environmental conditions) but such high temperatures remain scarce and most eels migrated at lower temperatures. A shift towards warmer temperature used is not surprising: both rivers are in the northern part of the species geographic range, where river temperatures are below the temperature optimum for eel growth^41,46^. When comparing silver European eels caught at different latitudes, growth rate tends to increase in the southern regions because of warmer temperatures^41,47^, although it has since been shown that this relationship appears to be more complex^48^. Increasing water temperature due to climate change is expected to increase eel growth in northern regions of Europe^46^, which includes the Imsa and Burrishoole rivers, and expand the geographic range of the European eel northward^49^. The hydrological regime may become the main restricting factor to eel migration, with summer discharges too low to trigger migration or requiring more energy resources for migrants. Along with this, the number of eels migrating in August in the Burrishoole River is positively related to the water level^29^. High summer discharge events have recently been more frequent and discharge variations may have stimulated a progressive early trigger of the migration on the Burrishoole River over the study period as observed by Sandlund et al.^18^, who have also proposed that this shift might be induced by a change in spring conditions leading to earlier silvering. In contrast, in the Mediterranean region, warm water temperatures combined with low discharges prevented silver eels from migrating during summer/fall and delayed migration until a water temperature drop in December/January^50^, several months later than in northern latitudes^16^. The contrasting trends in environmental conditions induced by global climate change is a real challenge for a panmictic species whose evolutionary capacities for local adaptation are limited^51^.

## Conclusion

The Imsa and Burrishoole rivers are located at different latitudes but have similar characteristics in terms of European eel subpopulations size and migration dynamics. We have developed a method to quantify the joint temporal trends in water temperature and discharge associations and their consequences on the available, effective and preferential niches of the downstream migration of European eel. Since the 1970s, silver eels have increasingly migrated during conditions of warm water temperatures (above 15 °C) associated with high discharge, a scenario which has become more frequent in the Imsa and Burrishoole rivers. So far, these conditions do not seem to have been limiting for the resilient European eel, but if the warming trend persists as expected in the coming decades, it will be very difficult to anticipate the practical consequences for the population and a mismatch between silvering “readiness” and the appropriate environmental window for migration could be feared. Besides, one could expect the environmental trends within the species geographic range to be even more heterogeneous than between the two watersheds studied, raising the issue of asynchronous migration between the northern and southern regions. Although we cannot definitively conclude on the impact of such environmental changes on the downstream migration of eels, we have broadly scanned the possible consequences, which may hold drastic implications for the persistence of an already critically endangered species. Importantly, we have developed a method that is particularly well suited to the analysis of long-term monitoring data and could be applied to non-panmictic species with a high homing rate (e.g., the Atlantic salmon *Salmo salar*), which may exhibit local adaptation and greater adaptability.

## Supplementary Information

Supplementary Information.

## Data Availability

Data available on request due to privacy/ethical restrictions: The data that support the findings of this study are available on request from the authors, OD and RP. The data are not publicly available due to private restrictions.
