# Supplementary material for: Joint temporal trends in river thermal and hydrological conditions can threaten the downstream migration of the critically endangered European eel
Source: Sci Rep. 2021 Aug 19;11:16927. doi: 10.1038/s41598-021-96302-x (PMC8377086; doi:10.1038/s41598-021-96302-x)
Supplement: Supplementary file 1 — Supplementary Information. [file 41598_2021_96302_MOESM1_ESM.docx]

Supplementary Material

**Joint temporal trends in river thermal and hydrological conditions can threaten the downstream migration of the critically endangered European eel**

Elorri Arevalo^1*^, Hilaire Drouineau^1^, Stéphane Tétard^2,3^, Caroline M.F. Durif^4^, Ola H. Diserud^5^, W. Russell Poole^6^, Anthony Maire^3^

^1^ INRAE, Unité EABX – Écosystèmes Aquatiques et Changements Globaux, HYNES (INRAE-EDF R&D), 50 avenue de Verdun, 33612 Cestas Cedex, France

^2^ ICEO Environnement, 52 Ter avenue des Sables, 85440 Talmont-Saint-Hilaire

^3^ EDF Recherche et Développement, Laboratoire National d’Hydraulique et Environnement, HYNES (INRAE-EDF R&D), 6 quai Watier, 78401 Chatou Cedex, France

^4^ Institute of Marine Research, Austevoll research station-Saugeneset 16, 5392, Storebø, Norway

^5^ Norwegian Institute for Nature Research, P.O. Box 5685, Torgarden 7485, Trondheim, Norway

^6^ Marine Institute, Furnace, Newport, Co. Mayo, Ireland

* Corresponding author: elorri.arevalo@gmail.com

Table S1 Summary statistics (mean ± SD) of water temperature (in °C) and discharge (in m^3^ s^-1^) or water level (in m) for the Imsa and Burrishoole rivers at annual and seasonal scales. JFM = January, February, March; AMJ = April, May, June; JAS = July, August, September; OND = October, November, December.

|  | Imsa River  Norway | Burrishoole River  Ireland |
| --- | --- | --- |
|  | Water temperature (°C) | Water temperature (°C) |
| Yearly | 9.4 ± 5.9 | 10.5 ± 4.4 |
| Winter (JFM) | 2.8 ± 1.0 | 5.5 ± 1.3 |
| Spring (AMJ) | 11.2 ± 4.7 | 11.1 ± 3.1 |
| Summer (JAS) | 16.6 ± 2.4 | 15.9 ± 1.7 |
| Fall (OND) | 7.0 ± 2.9 | 9.5 ± 2.6 |
|  |  |  |
|  | Discharge (m^3^ s^-1^) | Water level (m) |
| Yearly | 5.2 ± 4.1 | 0.41 ± 0.19 |
| Winter (JFM) | 6.6 ± 4.0 | 0.47 ± 0.17 |
| Spring (AMJ) | 2.9 ± 1.9 | 0.32 ± 0.15 |
| Summer (JAS) | 3.7 ± 3.2 | 0.35 ± 0.17 |
| Fall (OND) | 7.7 ± 4.5 | 0.50 ± 0.20 |

Table S2 Percentage of significant changes in the occurrence of water temperature and discharge/water level associations in (1) the available niche at annual and seasonal scales, (2) the available niche during the core migration period (from August to November), (3) the effective niche and (4) the preferential niche of silver eels to perform their downstream migration for the Imsa and Burrishoole rivers. Total percentage of significant changes and percentages of the associations that have become more or less frequent are shown (“Total”, “Gain” and “Loss”).

|  | Imsa River  Norway | | | Burrishoole River  Ireland | | |
| --- | --- | --- | --- | --- | --- | --- |
|  | Total | Gain | Loss | Total | Gain | Loss |
| Yearly | **32.4** | 28.0 | 4.4 | **16.8** | 14.2 | 2.6 |
| Winter (JFM) | **47.7** | 47.6 | 0.2 | **16.7** | 9.2 | 7.5 |
| Spring (AMJ) | **32.5** | 30.5 | 2.0 | **20.6** | 15.6 | 5.0 |
| Summer (JAS) | **24.8** | 14.4 | 10.4 | **8.8** | 7.0 | 1.8 |
| Fall (OND) | **24.0** | 18.3 | 5.7 | **30.1** | 24.7 | 5.4 |
| Available niche (from August to November) | **26.0** | 21.7 | 4.3 | **14.1** | 9.7 | 4.4 |
| Effective niche | **25.3** | 14.9 | 10.4 | **35.6** | 28.3 | 7.3 |
| Preferential niche | **23.6** | 8.6 | 15.0 | **36.4** | 24.1 | 12.3 |

Fig.S1 Monthly discharge (in m^3^ s^-1^) and water level (in m) for the Imsa and Burrishoole rivers, respectively. The bars correspond to the interannual average over the study period and the whiskers to the standard deviation.


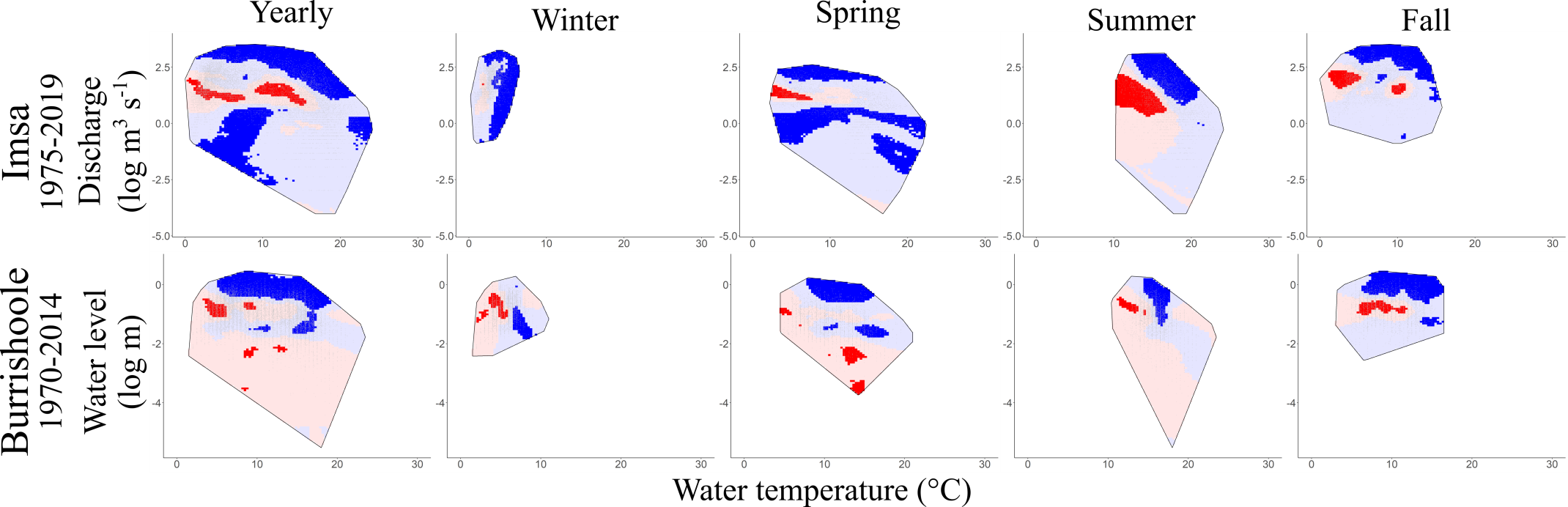


Fig.S2 Two-dimensional heatmaps of water temperature (X-axis; °C) and log-transformed discharge/water level (Y-axis; log m^3^ s^-1^ or log m) for the Imsa (top) and Burrishoole (bottom) rivers at the annual and seasonal scales (Winter: from January to March; Spring: from April to June; Summer: from July to September; Fall: from October to December). Water temperature × discharge/water level associations that have become more or less frequent over the study period are shown in blue or red, respectively. Light and dark colors correspond to non-significant and significant trends, respectively (e.g., dark red corresponds to significant decreasing trends, light blue to non-significant increasing trends). The gray line delineates the convex hull (*i.e.*, the smallest space encompassing all the points of the dataset, either annual or seasonal).


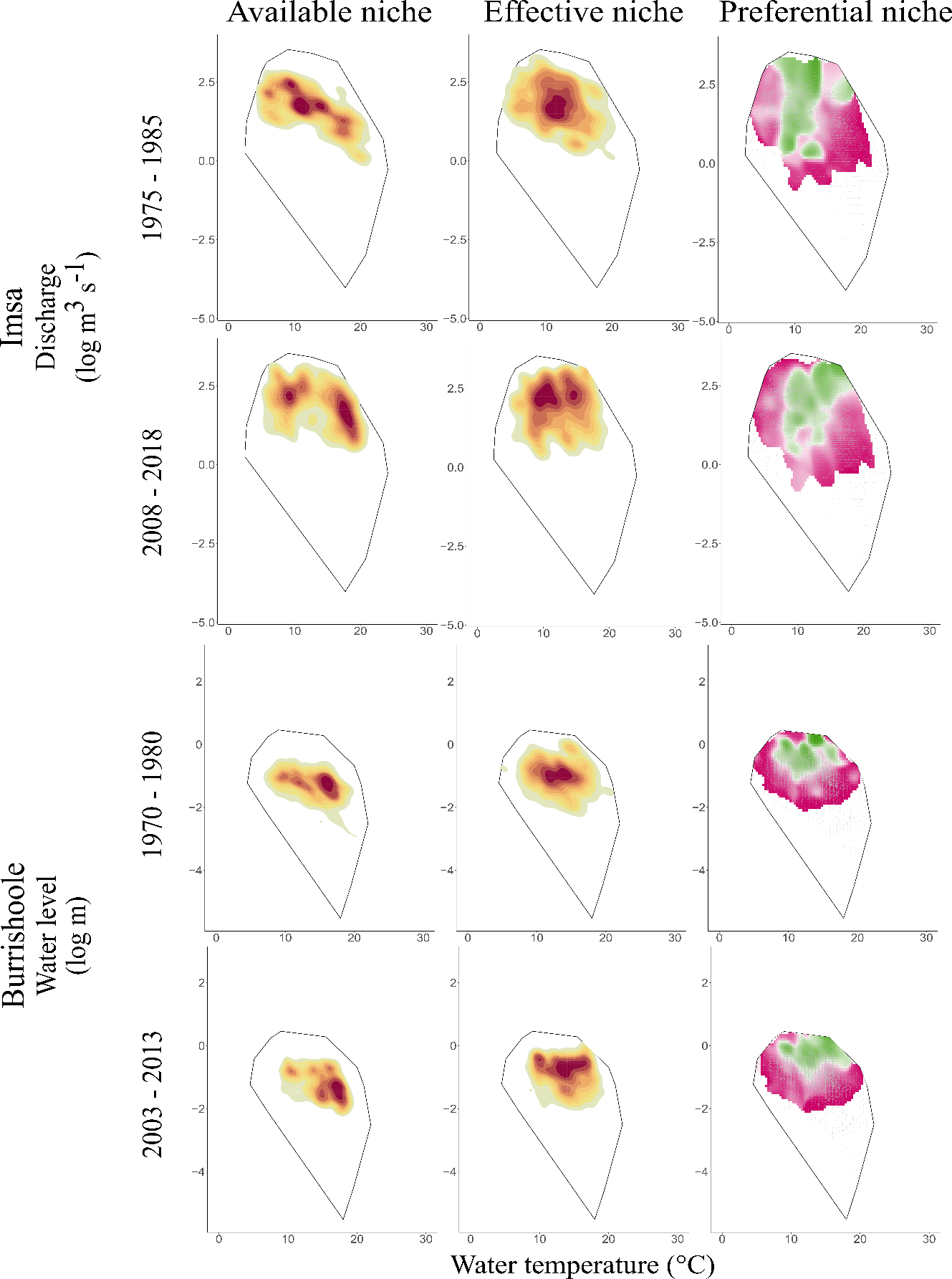


Fig.S3 Two-dimensional heatmaps of water temperature (X-axis; °C) and log-transformed discharge/water level (Y-axis; log m^3^ s^-1^ or log m) representing the frequency of the environmental associations (through kernel density estimations) within the available and effective niches and the environmental selection of silver eels within the preferential niche (through the Ivlev electivity index) at the beginning (first ten years) and at the end (last ten years) of the study period. For the available and effective niches, the colour scale ranges from dark red, which corresponds to the most frequent conditions (10% of the most frequent associations), to light yellow, which corresponds to the least frequent conditions (90% of the most frequent associations). Associations selected or rejected are shown in green or purple, respectively. The gray line delineates the convex hull computed at the annual scale over the entire study period.
